# A pilot study of maternal vitamin D status and its association with breast milk and infant gut metabolites

**DOI:** 10.14814/phy2.70774

**Published:** 2026-03-03

**Authors:** Alyssa S. Gutierrez, Katherine E. Chetta, Santosh Thapa, Sigmund J. Haidacher, Kathleen M. Hoch, John E. Baatz, Anthony M. Haag, Numan Oezguen, Thomas D. Horvath, Carol L. Wagner, Melinda A. Engevik

**Affiliations:** ^1^ Department of Regenerative Medicine & Cell Biology Medical University of South Carolina Charleston South Carolina USA; ^2^ Department of Pediatrics, C.P. Darby Children's Research Institute Medical University of South Carolina Charleston South Carolina USA; ^3^ Department of Pediatrics, Division of Neonatal‐Perinatal Medicine Medical University of South Carolina, Shawn Jenkins Children's Hospital Charleston South Carolina USA; ^4^ Department of Molecular Virology and Microbiology Baylor College of Medicine Houston Texas USA; ^5^ Texas Children's Research Institute Texas Children's Hospital Houston Texas USA; ^6^ Department of Pathology & Immunology Baylor College of Medicine Houston Texas USA; ^7^ Department of Pharmacy Practice and Translational Research, College of Pharmacy University of Houston Houston Texas USA; ^8^ Department of Pharmacology & Immunology Medical University of South Carolina Charleston South Carolina USA

**Keywords:** amino acids, breast milk, human milk, neurotransmitters, short‐chain fatty acids

## Abstract

Human milk contains multiple bioactive components, many of which are influenced by the mother's nutritional status. To identify the impact of maternal vitamin D status on neuroactive compounds, we conducted a post hoc analysis of breast milk and matched infant stool samples from mothers categorized as sufficient or deficient for vitamin D. Neuroactive metabolites were quantified using both targeted and nontargeted liquid chromatography with tandem mass spectrometry (LC‐MS/MS). Our findings revealed that breast milk from mothers with sufficient vitamin D levels contained significantly higher concentrations of tryptophan, phenylalanine, and tyrosine compared to milk from mothers with lower vitamin D levels. No significant differences were observed in tryptamine, kynurenine, kynurenic acid, anthranilic acid, quinolinic acid, tyramine, dopamine, epinephrine, or norepinephrine between the two groups. Among SCFAs and other acids, only hexanoic acid was significantly elevated in the breast milk of mothers with sufficient vitamin D. A nontargeted metabolomics analysis of infant stool identified distinct metabolite profiles, where oleamide, vaccenic acid, lacto‐N‐triaose, and N‐acetyl‐D‐glucosamine varied according to maternal vitamin D levels, indicating that maternal nutrient status may influence the infant gut metabolome. These findings suggest that maternal vitamin D status is associated with neurotransmitter precursor levels in breast milk and a distinct metabolomic profile of infant stool.

## Graphical abstract

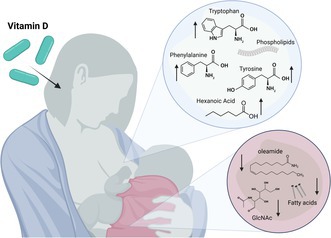

Vitamin D status in our pilot study was associated with higher levels of certain amino acids and hexanoic acid in breast milk and metabolites in infant stool samples.

## 1 INTRODUCTION

Human milk is a dynamic fluid that contains hundreds to thousands of bioactive molecules that benefit the infant (Ballard & Morrow, 2013; Brockway et al., 2024; Szyller et al., 2024). One bioactive compound found in human milk is vitamin D. There are two forms of vitamin D: cholecalciferol (vitamin D3) and ergocalciferol (vitamin D2). In adults, vitamin D sufficiency is usually achieved by sun exposure. In this setting, ultraviolet‐B (UV‐B) catalyzes the cutaneous conversion of 7‐dehydrocholesterol to vitamin D3. Adults also obtain small amounts of vitamin D through diet, as vitamin D3 is naturally in animal foods such as fatty fish and eggs or is supplemented in dairy products, and vitamin D2 is found in certain plants such as mushrooms (Rockwell et al., 2018). In contrast to adults, infants almost exclusively achieve vitamin D sufficiency through diet (Gallo et al., 2022). After consumption or endogenous synthesis of vitamin D3 and vitamin D2, the vitamin D is subsequently converted into calcidiol (25‐D) in the liver. 25‐D is the major circulating form of vitamin D and is therefore the targeted analyte for assessing an individual's vitamin D status. Finally, 25‐D is converted in the kidneys to calcitriol (1,25‐D), which is the biologically active form of vitamin D.

Despite diet being the primary source of vitamin D for the nursing infant, most breast milk lacks sufficient vitamin D levels to support the nutritional needs of the developing infant. For this reason, the American Academy of Pediatrics (AAP) and the Institute of Medicine (IOM) strongly recommend vitamin D supplementation for all breastfed infants (Institute of Medicine (US) Standing Committee on the Scientific Evaluation of Dietary Reference Intakes, 1997; Hollis et al., 2015; Wagner & Greer, 2008). While direct supplementation to infants is now a common practice, our group has previously shown that breastfed infants can indirectly achieve vitamin D sufficiency if lactating mothers are supplemented with high levels of vitamin D3 (Hollis et al., 2015; Hollis & Wagner, 2004; Wagner et al., 2006, 2020). Vitamin D has been demonstrated to be very important during early life development (Berridge, 2015; Bikle, 2011; Vitamin, 2006). Several studies have associated adequate vitamin D levels during both fetal and neonatal life with lower risks of wheezing, chronic lung diseases such as bronchopulmonary dysplasia, atopic diseases such as asthma, and some allergies in early childhood (Al‐Beltagi et al., 2020; Belderbos et al., 2011; Hansdottir et al., 2010; Hibbs et al., 2018; Hollams et al., 2011; Jones et al., 2015; Park et al., 2020). More recently, vitamin D has garnered interest for its role in neurodevelopment, particularly in regulating neurotransmitter systems (De Marzio et al., 2024; Rodgers et al., 2023; Yasumitsu‐Lovell, Thompson, Fernell, Eitoku, Suganuma, Gillberg, Environment, & Group, 2024; Ye et al., 2023). Vitamin D signaling was shown to play a supportive role in dopaminergic and glutaminergic system development and functions (Kasatkina et al., 2020; Kesby et al., 2017; Pertile et al., 2016, 2023). Additionally, vitamin D deficiency in children has been associated with the development of autism spectrum disorder (ASD), attention‐deficit/hyperactivity disorder (ADHD), and decreased cognitive function (De Marzio et al., 2024; Kotsi et al., 2019; Rodgers et al., 2023; Yasumitsu‐Lovell et al., 2024). Vitamin D supplementation has been shown to modulate the fecal microbiota, and additional data suggest that vitamin D signaling within the gut microbiome could be associated with ASD and ADHD (Ma et al., 2022; Ogbu et al., 2020).

Given the importance of vitamin D signaling in early life neurodevelopment, we sought to investigate the influence of vitamin D sufficiency on neurotransmitters in breast milk and infant feces. Little is known about the effects of maternal vitamin D status on levels of neuroactive compounds and neurotransmitters in breast milk. Likewise, little is known about how vitamin D levels in breast milk might affect the abundance of neuroactive compounds and neurotransmitters in the infant gut. To address these questions, we studied six mother‐infant dyads grouped by sufficient or deficient vitamin D status. We defined vitamin D status based on the National Institute of Health (NIH) guidelines, where serum 25‐D concentrations of ≥20 ng/mL (50 nmol/L) are sufficient and levels below that threshold are deficient (National Institutes of Health, 2025). Three mother–child dyads were defined as deficient with maternal serum 25‐D < 20 ng/mL, while the other three dyads were defined as sufficient with maternal serum 25‐D > 45 ng/mL. We examined breast milk samples and matched infant stool samples by targeted and nontargeted liquid chromatography–tandem mass spectrometry (LC‐MS/MS) based bioanalysis. We hypothesized that vitamin D‐sufficient mother‐infant dyads would exhibit distinct breast milk and infant stool neurotransmitter and neuroactive metabolite profiles compared to vitamin D‐deficient dyads.

## 2 MATERIALS AND METHODS

### 2.1 Ethical approval and study design

This study was approved by the Medical University of South Carolina (PRO #0050609), and the methods have been previously reported (Hollis et al., 2015; Newton et al., 2022). Briefly, exclusively breastfeeding mothers in good general health and their healthy 4‐ to 6‐week‐old infants (≥35 weeks' gestation) were enrolled in the parent trial (Hollis et al., 2015), and written consent was obtained for participation. In this study, mothers were randomized to either placebo or 400, 2400, or 6400 IU vitamin D3/day (Tishcon Corporation, Westbury, NY) for 3 months. No other vitamins were supplemented. Most subjects in the parent trial were identified as non‐Hispanic Caucasian or Caucasian.

### 2.2 Sample selection

Given that the authors still are blinded to assigned study groups from the larger clinical trial, the samples were selected based on 3‐month circulating maternal vitamin D levels and were not directly related to the supplementation in the larger clinical trial. A total of six mother–child dyads were selected based on serum vitamin D levels. According to the NIH, vitamin D sufficiency can be confirmed by serum 25‐D concentrations of ≥20 ng/mL (50 nmol/L) (National Institutes of Health, 2025). Three dyads were selected by sufficient maternal serum vitamin D (25‐D > 45 ng/mL), while the other three dyads were chosen by deficient maternal serum vitamin D (25‐D < 20 ng/mL). At the 3‐month follow‐up visit, the mother expressed breast milk in a hygienic fashion into a container as per study protocol, and an infant stool was collected from a diaper. Samples were freshly collected in the early morning and stored during the AM research clinic visit. Breast milk (*n* = 6) and stool samples (*n* = 6) were collected from mother–child dyads. We analyzed feces from 2 female and 1 male infant from mothers with sufficient vitamin D and 2 male and 1 female infants from mothers with insufficient vitamin D status.

### 2.3 Breast milk and infant stool sample extraction for LC–MS/MS

All samples were processed using ice‐cold methanol. Breast milk samples were treated with an equal volume of ice‐cold methanol (1:1 *v/v* methanol). Tubes were centrifuged at 12,000 x *g* for 5 min to pellet any solids, and the cell‐free supernatant was used for downstream analysis. This extraction step results in phase separation, with the lipid layer forming at the top of the tube; only the clarified methanolic aqueous supernatant was collected and used for downstream LC‐MS/MS analysis, while the lipid layer was not analyzed. Infant stool samples were weighed, and 100 mg of feces was added into 2 mL fast prep‐tubes (MP Biomedical #MP115076200) harboring 0.1 g of 1.4 mm ceramic beads (MP Biomedical Lysing Matrix D #116540434), similar to previously published work (Engevik et al., 2024). To each tube, a 1 mL volume of ice‐cold methanol and water (1:1, *v/v*) was added, and the tubes were homogenized on a Benchmark “Beadbug” 6 Microtube Homogenizer (Stellar Scientific, #BS‐BEBU‐6) at 4.0 m/s for 20 seconds for two cycles. Tubes were centrifuged at 12,000 x *g* for 5 min to pellet any solids, and the cell‐free supernatant was used for downstream analysis.

### 2.4 Reagents and chemicals

Optima™ LC/MS‐grade water, acetonitrile (Fisher Scientific, cat. no. A955‐4), methanol (Fisher Scientific, cat. no. A456‐4), and MS‐grade formic acid (Fisher Scientific, cat. no. A117‐50), and acetic acid (Fisher Scientific, cat. no. A11350) were obtained from ThermoFisher Scientific (Waltham, MA, USA). MS‐grade ammonium formate (Millipore‐Sigma, cat. no. 70221), ammonium acetate (Millipore‐Sigma, cat. no. 73594), and heptafluorobutyric acid (HFBA) (Millipore‐Sigma, cat. no. 52411) were purchased from Millipore‐Sigma (Burlington, MA, USA). Chemicals used in the SCFA derivatization and quenching procedures, including 1‐(3‐dimethylaminopropyl)‐3‐ethylcarbodiimide hydrochloride (EDAC) (Fisher Scientific, cat. no. 03450), 2‐mercaptoethanol (Fisher Scientific, cat. no. AC125470100), and succinic acid (Fisher Scientific, cat. no. AC158742500) were all purchased from Fisher Scientific (Waltham, MA, USA). Unlabeled aniline (Millipore‐Sigma, cat. no. 132934) and [^13^C_6_]‐aniline for SCFA method internal standard (IS) (Millipore‐Sigma, cat. no. 485497) preparations were purchased from Millipore Sigma.

### 2.5 Analytical standards, internal standards, and analytical columns used for quantitative analysis

The targeted Tryptophan Pathway method covers portions of the hydroxylation (serotonin production), decarboxylation (tryptamine production), and transamination (indoleacetic acid production) pathways for tryptophan metabolism. Analytical standards for tryptophan (Fisher Scientific, cat. no. AC140590250), tryptamine hydrochloride (Millipore‐Sigma, cat. no. 246557), serotonin hydrochloride (Fisher Scientific, cat. no. AC215025000), melatonin (Fisher Scientific, cat. no. ICN10225401), and 5‐hydroxytryptophan (Fisher Scientific, cat. no. H05311G) were purchased from Fisher Scientific. N‐acetylserotonin (Millipore‐Sigma, cat. no. A1824), indoleacetic acid (Millipore‐Sigma, cat. no. I3750), and 5‐hydroxyindole‐3‐acetic acid (5‐HIAA) (Millipore‐Sigma, cat. no. H8876) standards were purchased from Millipore‐Sigma. The d_5_‐5‐HIAA (IS) (CDN Isotopes, cat. no. D‐6725), d_3_‐5‐hydroxytryptophan (IS) (CDN Isotopes, cat. no. D‐7154), d_4_‐melatonin (CDN Isotopes, cat. no. D‐2952), d_5_‐L‐tryptophan (IS) (CDN Isotopes, cat. no. D‐1522) compounds were purchased from CDN Isotopes (Pointe‐Claire, Quebec, Canada), and the d_4_‐serotonin hydrochloride (IS) (Santa Cruz Biotechnology, cat. no. sc‐473411) standard was purchased from Santa Cruz Biotechnology (Dallas, TX, USA). This combined tryptophan hydroxylation, decarboxylation, and transamination pathway method separation was performed using a 3‐micron Luna C18(2) (150 mm × 1 mm; 100 Å pore) analytical column equipped with a Security Guard C18 (4 mm × 2 mm) guard column that were purchased from Phenomenex (Torrance, CA USA).

The targeted Tryptophan‐Kynurenine Pathway method includes analytical standards for phenylalanine (Millipore‐Sigma, cat. no. P2126), kynurenine (Millipore‐Sigma, cat. no. K8625), 3‐hydroxykynurenine (Millipore‐Sigma, cat. no. H1771), kynurenic acid (Millipore‐Sigma, cat. no. 94539), and indole (Millipore‐Sigma, cat. no. I3408) that were all purchased from Millipore‐Sigma. The d_7_‐indole (IS) (CDN Isotopes, cat. no. D‐1897), d_6_‐kynurenine (IS) (Cambridge Isotope Laboratories, cat. no. DLM‐7842‐0.005), and d_5_‐phenylalanine (IS) (CDN Isotopes, cat. no. D‐1597) compounds were purchased from CDN Isotopes. This targeted Tryptophan‐Kynurenine Pathway method separation was performed using a 3‐micron Luna C18(2) (150 mm x 1 mm; 100 Å pore) analytical column equipped with a Security Guard C18 (4 mm x 2 mm) guard column that was purchased from Phenomenex.

For the tyrosine pathway method, the tyrosine (Millipore‐Sigma, cat. no. T3754), D,L‐norepinephrine (Millipore‐Sigma, cat. no. A7256), Levodopa (L‐DOPA) (Millipore‐Sigma, cat. no. D9628), d_3_‐L‐DOPA (IS) (Millipore‐Sigma, cat. no. 333786), epinephrine (Millipore‐Sigma, cat. no. E4250), d_6_‐epinephrine (IS) (Millipore‐Sigma, cat. no. E‐077), dopamine hydrochloride (Millipore‐Sigma, cat. no. H8502), d_4_‐dopamine (IS) (Millipore‐Sigma, cat. no. 655651), and anthranilic acid (Millipore‐Sigma, cat. no. 10680) standards were all purchased from Millipore‐Sigma. The L‐tyramine (Fisher Scientific, cat. no. AC140610250) and d_4_‐L‐tyramine (IS) (Santa Cruz Biotechnology, cat. no. sc‐220347) standards were purchased from Fisher Scientific and Santa Cruz Biotechnology, respectively. The Tyrosine Pathway separation was performed using a 2.7‐micron Raptor C18 (100 mm × 1 mm; 90 Å pore) analytical column equipped with a 5‐micron Ultra C18 (4 mm × 2 mm) guard column that were purchased from Restek.

For the Glutamate Cycle method, the glutamate (Millipore‐Sigma, cat. no. G1251), glutamine (Millipore‐Sigma, cat. no. G3126), GABA, d_6_‐GABA (IS) (Millipore‐Sigma, cat. no. 615587), and d_5_‐L‐glutamate (IS) (Millipore‐Sigma, cat. no. 616281) were all purchased from Millipore‐Sigma, and d_5_‐L‐glutamine (IS) (CDN Isotopes, cat. no. D‐2532) standard was purchased from CDN Isotopes (Pointe‐Claire, Quebec, Canada). The Glutamate Cycle method separation was performed using a 2.7‐micron Supelco Ascentis Express hydrophilic interaction chromatography (HILIC) (150 × 2.1 mm; 90 Å pore) analytical column that was purchased from Millipore‐Sigma.

For the Trimethylamine‐N‐oxide (TMAO) Pathway method, the acetyl‐carnitine (Millipore‐Sigma, cat. no. A1509), free carnitine (Millipore‐Sigma, cat. no. C9500), betaine (Millipore‐Sigma, cat. no. 61962), choline (Millipore‐Sigma, cat. no. C7017), TMAO (Millipore‐Sigma, cat. no. 317594), acetylcholine (Millipore‐Sigma, cat. no. A6625) analytical standards, and the N‐methyl‐d_3_‐acetylcarnitine (IS) standard (Millipore‐Sigma, cat. no. 729884) were all purchased from Millipore‐Sigma. The TMAO Pathway method separation was performed using a 5‐micron polymeric SeQuant® ZIC®‐pHILIC (150 × 2.1 mm) analytical column that was purchased from Millipore‐Sigma.

For the SCFA method, an Optima™ LC/MS‐grade acetic acid standard was purchased from Fisher Scientific. Organic acid standards for propionic acid (Millipore‐Sigma, cat. no. 402907), isobutyric acid (Millipore‐Sigma, cat. no. I1754), butyric acid (Millipore‐Sigma, cat. no. B103500), 2‐methylbutyric acid (Millipore‐Sigma, cat. no. W269514), isovaleric acid (Millipore‐Sigma, cat. no. 129542), valeric acid (Millipore‐Sigma, cat. no. 240370), and hexanoic acid (Millipore‐Sigma, cat. no. 153745) were all purchased from Millipore‐Sigma. The SCFA method separation was performed using a 5.0‐micron Viva Biphenyl (100 × 1 mm; 300 Å pore) analytical column equipped with a 5.0‐micron Viva Biphenyl (10 × 2.1 mm) guard column that was purchased from Restek (Bellefonte, PA).

### 2.6 Critical solution and final sample preparations for the targeted tryptophan pathway, tyrosine pathway, and glutamate cycle methods

The procedures for the preparation of the critical solutions consisting of the analytical and IS stocks, combined intermediates, calibration standards (calibrators), and working Internal Standard Solutions (ISSs) for the targeted Tryptophan Pathway, Tyrosine Pathway, and Glutamate Cycle methods have been described previously (Engevik, Luck, et al., 2021; Horvath et al., 2022, 2023; Luck et al., 2021). The final sample preparation procedures for the targeted Tryptophan Pathway, Tyrosine Pathway, and Glutamate Cycle methods included a dilution of a 10‐μL volume of each individual maternal breast milk or infant fecal sample extract in a 90‐μL volume of the ISS‐A solution for the respective method directly in an autosampler vial, and the 10‐fold diluted sample was vortex‐mixed for 30 s. The samples were then transferred to the autosampler module, and a 5‐μL sample volume was injected onto the LC‐MS/MS system for analysis.

### 2.7 Synthetic procedures to produce the SCFA‐based N‐phenyl anilide analytical and internal standards

The procedures used to synthesize the unlabeled and [^13^C_6_]‐labeled N‐phenyl anilide based SCFA derivatives for the production of unlabeled analytical and [^13^C_6_]‐labeled IS stock solutions were described previously (Engevik, Luck, et al., 2021; Horvath et al., 2022, 2023; Luck et al., 2021). Individual IS stock solutions for [^13^C_6_]‐N‐phenylformamide (formic acid derivative IS), [^13^C_6_]‐N‐phenylacetamide (acetic acid derivative IS), [^13^C_6_]‐N‐phenylpropanamide (propionic acid derivative IS), [^13^C_6_]‐N‐phenylisobutanamide (isobutyric acid derivative IS), [^13^C_6_]‐N‐phenylbutanamide (butyric acid derivative IS), [^13^C_6_]‐N‐phenyl‐2‐methylbutanamide (2‐methylbutyric acid derivative IS), [^13^C_6_]‐N‐phenylisopentanamide (isovaleric acid derivative IS), [^13^C_6_]‐N‐phenylpentanamide (valeric acid derivative IS), and [^13^C_6_]‐N‐phenylhexanamide (hexanoic acid derivative IS) were each prepared at individual concentrations of 3.84 mM in a solution of acetonitrile: water (2:8, *v:v*) during the derivatization reaction. Unlabeled stock solutions of N‐phenylformamide (unlabeled formic acid derivative), N‐phenylacetamide (unlabeled acetic acid derivative), N‐phenylpropanamide (unlabeled propionic acid derivative), N‐phenylisobutanamide (unlabeled isobutyric acid derivative), N‐phenylbutanamide (unlabeled butyric acid derivative), N‐phenyl‐2‐methylbutanamide (unlabeled 2‐methylbutyric acid derivative), N‐phenylisopentanamide (unlabeled isovaleric acid derivative), N‐phenylpentanamide (unlabeled valeric acid derivative), and N‐phenylhexanamide (unlabeled hexanoic acid derivative) were each prepared at individual concentrations of 3.84 mM in a solution of acetonitrile: water (2:8, v:v) during the derivatization reaction.

### 2.8 Critical solution and final sample preparations for the targeted SCFA method

The procedures for the preparation of the critical solutions consisting of the combined intermediates, calibrators and working ISSs for the targeted SCFA method have been described previously (Engevik, Luck, et al., 2021; Horvath et al., 2022, 2023; Luck et al., 2021). The derivatization procedure for the extracted breast milk samples was performed by diluting a 35‐μL volume of each sample extract with a 35‐μL volume of an acetonitrile: water (1:1, *v:v*) solution. Then a 20‐μL volume of a freshly prepared aniline solution (100 mM concentration in acetonitrile: water (1:1, *v:v*)) was added to each sample. Finally, a 10‐μL volume of a freshly prepared 1‐(3‐dimethylaminopropyl)‐3‐ethylcarbodiimide hydrochloride (EDAC) solution (100 mM concentration in acetonitrile: water (1:1, *v:v*)) was added to each sample followed by brief vortex‐mixing. At this stage, the derivatization reaction for each sample was allowed to progress over a two‐hour incubation period at a temperature of +4°C. After completion of the derivatization reaction, individual volumes of 18.8‐μL and 12‐μL of freshly prepared succinic acid (250 mM in water) and 2‐mercaptoethanol (100 mM in water), respectively, were added into each sample to quench the reaction. The quenching reaction for each sample was allowed to progress for an additional two‐hour incubation period at a temperature of +4°C—the overall dilution factor (DF) up to this point was 3.74‐fold. A 10‐μL volume of the derivatized, maternal breast milk or infant fecal sample extract was diluted in a 90‐μL volume of the SCFA Method ISS‐A solution directly in an autosampler vial, and the 10‐fold diluted sample (DF = 37.4 overall) was vortex‐mixed for 30 s. The samples were transferred to the autosampler module and a 5‐μL sample volume was injected onto the LC‐MS/MS system for analysis.

### 2.9 Critical solution and final sample preparations for the tryptophan‐kynurenine pathway method

Individual IS stock solutions were prepared for d_7_‐indole, d_6_‐kynurenine, and d_5_‐phenylalanine at a 10.0 mg/mL concentration for each in a solution consisting of methanol: water (1:1, *v:v*). An ISS‐A solution was then created from these stocks, with concentrations of 250 ng/mL for each IS compound in water. A subsequent ISS‐B solution was prepared by diluting a 9 mL volume of the ISS‐A solution with 1 mL volume of water, resulting in concentrations of 225 ng/mL for each IS compound in water. All IS stocks were stored frozen at −80°C, and the ISS‐A and ISS‐B solutions were stored refrigerated (4°C) when not in use. Individual unlabeled stock solutions for indole, kynurenine, phenylalanine, 3‐hydroxykynurenine, and kynurenic acid were each prepared at 10.0 mg/mL in water. A combined intermediate was prepared from each of the stocks at a concentration of 100 μg/mL for each metabolite using the Tryptophan‐Kynurenine Pathway ISS‐B as the diluent. Calibrators were prepared from the combined intermediate by serial dilution (DF 4‐fold per Calibrator level) at metabolite concentrations of 1000, 250, 62.5, 15.6, 3.90, and 0.977 ng/mL using the Tryptophan‐Kynurenine Pathway ISS‐B as the diluent. The unlabeled stock solutions, the Combined Intermediate solution, and all Calibrators prepared for the Tryptophan‐Kynurenine Pathway method were stored frozen at −80°C when not in use. A 10‐μL volume of each individual maternal breast milk or infant fecal sample extract was diluted in a 90‐μL volume of the Tryptophan‐Kynurenine Pathway ISS‐A solution directly in an autosampler vial, and the 10‐fold diluted sample was vortex‐mixed for 30 s. The samples were transferred to an autosampler module and a 5‐μL sample volume was injected onto the LC‐MS/MS system for bioanalysis.

### 2.10 Critical solution preparations for the trimethylamine N‐oxide (TMAO) pathway method

An individual IS stock solution was prepared for N‐methyl‐d_3_‐acetylcarnitine at a 10.0 mg/mL concentration in water. An ISS‐A solution was then created from this stock at an N‐methyl‐d_3_‐acetylcarnitine concentration of 250 ng/mL in a solution consisting of acetonitrile: water (95/5) and 1% acetic acid. The IS stock was stored frozen at −80°C, and the ISS‐A solution was stored refrigerated (4°C) when not in use. Individual unlabeled stock solutions for TMAO, free carnitine, acetylcarnitine, choline, betaine, and acetylcholine were each prepared at 10.0 mg/mL in water. A combined intermediate was prepared from each of the stocks at a concentration of 50 μg/mL for each metabolite using the TMAO Pathway ISS‐A as the diluent. Calibrators were prepared from the combined intermediate by serial dilution (DF 4‐fold per Calibrator level) at metabolite concentrations of 500, 125, 31.2, 6.25, 1.56, and 0.39 ng/mL using the TMAO Pathway ISS‐A as the diluent. The unlabeled stock solutions, the combined intermediate solution, and all calibrators prepared for the TMAO Pathway method were stored frozen at −80°C when not in use. A 10‐μL volume of each individual maternal breast milk or infant fecal sample extract was diluted in a 190‐μL volume of the TMAO Pathway ISS‐A solution directly in an autosampler vial, and the 20‐fold diluted sample was vortex‐mixed for 30 s. The samples were transferred to an autosampler module and a 1‐μL sample volume was injected onto the LC‐MS/MS system for bioanalysis.

### 2.11 LC‐MS/MS equipment for targeted and nontargeted metabolomics based bioanalysis

The LC‐MS/MS system used for the targeted bioanalysis consisted of a Nexera X2 Ultrahigh‐Performance Liquid Chromatography (UHPLC) system (Shimadzu, Kyoto, Japan) connected to a QTRAP 6500 hybrid triple‐quadrupole/linear ion trap mass spectrometer (SCIEX, Framingham, MA, USA). The system was operated using Analyst® software (Version 1.6.2; SCIEX), while peak integration and quantitative analysis were performed using the MultiQuant™ software (Version 3.0.1; SCIEX).

The high‐resolution LC‐MS/MS system used for nontargeted bioanalysis was comprised of a Nexera X2 UHPLC system connected to an Orbitrap Fusion™ Tribrid™ MS system (ThermoFisher Scientific, Waltham, MA USA). Operations of the individual UHPLC and Orbitrap Fusion systems were controlled by LabSolutions software (Ver. 5.8.2; Shimadzu) and Xcalibur software (Ver. 4.3.73.11; ThermoFisher Scientific), respectively. Raw data files were imported into the Proteome Software Scaffold Elements (Ver. 3.0.3; ProteoWizard) software for final analysis.

### 2.12 Targeted LC‐MS/MS method for the targeted tryptophan pathway, tyrosine pathway, glutamate cycle, and SCFA methods

The targeted LC‐MS/MS methods used on the SCIEX QTRAP 6500 for the tryptophan pathway, tyrosine pathway, glutamate cycle, and SCFA methods have been described previously (Engevik, Luck, et al., 2021; Horvath et al., 2022, 2023; Luck et al., 2021). The metabolite specific SRM transition parameters for the targeted tryptophan pathway, tyrosine pathway, glutamate cycle, and the SCFA method are contained in the Tables 1, 2, 3, 4, respectively.

**TABLE 1:** Selected‐reaction monitoring (SRM) transition parameters for the tryptophan pathway metabolites on the Sciex 6500 QTrap MS.

Metabolite	Q1 (*m/z*)	Q3 (*m/z*)^a^	DP (V)	EP (V)	CE (eV)	CXP (V)
Tryptophan	205.1	188.1/146.1	35	7	15/25	15
d_5_‐tryptophan (IS)	210.1	192.1/150.1	35	7	15/25	15
Serotonin	177.1	160.1/115.1	20	8	17/38	10
d_4_‐serotonin (IS)	181.1	164.1/119.1	20	8	17/38	10
Melatonin	233.1	174.1/159.1	45	6	22/39	15
d_4_‐melatonin (IS)	237.1	178.1/163.1	45	6	22/39	15
5‐hydroxy‐indoleacetic acid (5‐HIAA)	192.1	146.1/118.1	70	8	23/40	17
d_5_‐5‐HIAA (IS)	197.1	150.1/122.1	70	8	23/40	17
5‐hydroxy‐tryptophan	221.1	204.1/162.1	40	7	16/26	23
N‐acetylserotonin	219.1	160.1/115.1	40	8	23/49	10
Tryptamine	161.1	144.1/115.1	30	9	18/49	20
Indole‐3‐acetic acid	176.1	130.1/77.0	70	4	25/56	11

Abbreviations: CE, collision energy; CXP, collision‐cell exit potential; DP, declustering potential; EP, entrance potential; eV, electron volts; V, volts.

^a^ The black text corresponds to the mass‐to‐charge (*m/z*) of the quantifying fragment ion, and the red text corresponds to the *m/z* of the qualifying fragment ion.

**TABLE 2:** Selected‐reaction monitoring (SRM) transition parameters for the Tyrosine Pathway metabolites on the Sciex 6500 QTrap MS.

Metabolite	Q1 (*m/z*)	Q3 (*m/z*)^a^	DP (V)	EP (V)	CE (eV)	CXP (V)
Tyrosine	182.1	165.1/136.1	50	8	14/19	10
Tyramine	138.1	121.1/103.1	40	8	16/29	10
d_4_‐tyramine (IS)	142.1	125.1/106.1	40	8	16/29	10
dopamine	154.1	137.1/91.1	40	9	15/32	10
d_4_‐dopamine (IS)	158.1	141.1/95.1	40	9	15/32	10
L‐DOPA	198.1	181.1/152.1	50	7	14/20	9
d_3_‐L‐DOPA (IS)	201.1	184.1/155.1	50	7	14/20	9
Norepinephrine	170.1	152.1/107.1	40	9	12/26	9
Epinephrine	184.1	166.1/151.1	40	8	15/30	15
d_6_‐epinephrine (IS)	190.1	172.1/157.1	40	8	15/30	15
Anthranilic acid	138.0	120.0/92.0	30	4	16/38	12
Quinolinic acid	168.0	150.0/78.0	40	3	14/30	18

Abbreviations: CE, collision energy; CXP, collision‐cell exit potential; DP, declustering potential; EP, entrance potential; eV, electron volts; V, volts.

^a^ The black text corresponds to the mass‐to‐charge (*m/z*) of the quantifying fragment ion, and the red text corresponds to the *m/z* of the qualifying fragment ion.

**TABLE 3:** SRM transition parameters for the Glutamate Cycle metabolites on the SCIEX QTRAP 6500 MS system.

Metabolite	Q1 (*m/z*)	Q3 (*m/z*)^a^	DP (V)	EP (V)	CE (eV)	CXP (V)
GABA	104.1	87.1/69.1	80	9	20/30	9
d_6_‐GABA (IS)	110.1	92.1/73.1	80	9	20/30	9
Glutamate	148.1	84.0/130.1	55	9	24/14	11
d_5_‐glutamate (IS)	153.1	88.1/135.1	55	9	24/14	11
Glutamine	147.1	130.1/84.1	55	9	14/24	11
d_5_‐glutamine (IS)	152.1	135.1/88.1	55	9	14/24	11

Abbreviations: CE, collision energy; CXP, collision‐cell exit potential; DP, declustering potential; EP, entrance potential; eV, electron volts; V, volts.

^a^ The black text corresponds to the mass‐to‐charge (*m/z*) of the quantifying fragment ion, and the red text corresponds to the *m/z* of the qualifying fragment ion.

**TABLE 4:** SRM transition parameters for the N‐phenyl SCFA derivatives on the SCIEX QTRAP 6500 MS system.

Metabolite	Q1 (*m/z*)	Q3 (*m/z*)^a^	DP (V)	EP (V)	CE (eV)	CXP (V)
N‐phenyl formamide	122.1	94.0/77.0	80	8	22/32	15
[^13^C_6_]‐N‐phenyl formamide (IS)	128.1	100.0/83.0	70	8	21/35	15
N‐phenyl acetamide	136.1	94.0/77.0	60	9	22/40	15
[^13^C_6_]‐N‐phenyl acetamide (IS)	142.1	100.0/83.0	60	10	22/51	15
N‐phenyl propanamide	150.1	94.0/77.0	70	9	22/42	15
[^13^C_6_]‐N‐phenyl propanamide (IS)	156.1	100.0/83.0	60	10	26/45	15
N‐phenyl butanamide^b^	164.1	94.0/77.0	75	9	23/45	15
[^13^C_6_]‐N‐phenyl butanamide (IS)	170.1	100.0/83.0	60	10	25/49	15
N‐phenyl isobutanamide^b^	164.1	94.0/77.0	72	9	24/46	15
[^13^C_6_]‐N‐phenyl isobutanamide (IS)	170.1	100.0/83.0	72	9	24/46	15
N‐phenyl 2‐methylbutanamide^c^	178.1	94.0/77.0	80	9	23/49	15
[^13^C_6_]‐N‐phenyl 2‐methylbutanamide (IS)	184.1	100.0/83.0	80	9	23/49	15
N‐phenyl pentanamide^c^	178.1	94.0/77.0	80	9	23/48	15
[^13^C_6_]‐N‐phenyl pentanamide (IS)	184.1	100.0/83.0	80	9	23/48	15
N‐phenyl isopentanamide^c^	178.1	94.0/77.0	80	9	23/49	15
[^13^C_6_]‐N‐phenyl isopentanamide (IS)	184.1	100.0/83.0	80	9	23/49	15
N‐phenyl hexanamide	192.1	94.0/77.0	90	9	26/54	15
[^13^C_6_]‐N‐phenyl hexanamide (IS)	198.1	100.0/83.0	90	8	26/55	15

Abbreviations: CE, collision energy; CXP, collision‐cell exit potential; DP, declustering potential; EP, entrance potential; eV, electron volts; V, volts.

^a^ The black text corresponds to the mass‐to‐charge (*m/z*) of the quantifying fragment ion, and the red text corresponds to the *m/z* of the qualifying fragment ion.

^b^ N‐phenyl butanamide and N‐phenyl isobutanamide, and their respective IS materials, are isobaric compounds and are resolved chromatographically.

^c^ N‐phenyl 2‐methylbutanamide, N‐phenyl pentanamide, and N‐phenyl isopentanamide and their respective IS materials are all isobaric compounds and are resolved chromatographically.

**TABLE 5:** Selected‐reaction monitoring (SRM) transition parameters for the Tryptophan‐Kynurenine Pathway metabolites on the Sciex 6500 QTrap MS.

Metabolite	Q1 (*m/z*)	Q3 (*m/z*)^a^	DP (V)	EP (V)	CE (eV)	CXP (V)
Indole	118.1	91.1/65.0	100	8	30/46	10
d_7_‐Indole (IS)	124.1	96.1/69.0	100	8	40/46	10
Kynurenine	209.1	192.1/94.1	20	8	14/20	10
d_6_‐Kynurenine (IS)	215.1	198.1/98.1	20	8	14/20	10
Phenylalanine	166.1	120.1/103.0	40	8	23/40	10
d_5_‐Phenylalanine (IS)	171.1	125.1/107.0	40	8	23/40	10
3‐Hydroxykynurenine	225.1	162.0/110.0	40	8	29/25	10
Kynurenic Acid	190.0	144.0/162.0	60	8	29/24	10

Abbreviations: CE, collision energy; CXP, collision‐cell exit potential; DP, declustering potential; EP, entrance potential; eV, electron volts; V, volts.

^a^ The black text corresponds to the mass‐to‐charge (*m/z*) of the quantifying fragment ion, and the red text corresponds to the *m/z* of the qualifying fragment ion.

**TABLE 6:** Selected‐reaction monitoring (SRM) transition parameters for the trimethylamine‐N‐oxide pathway metabolites on the Sciex 6500 QTrap MS.

Metabolite	Q1 (*m/z*)	Q3 (*m/z*)^a^	DP (V)	EP (V)	CE (eV)	CXP (V)
TMAO	76.0	58.0/42.0	100	10	26/49	15
Free Carnitine	162.1	85.0/60.0	60	10	28/23	15
Acetylcarnitine	204.1	85.0/60.0	60	10	28/20	15
Choline	104.1	60.0/58.0	100	10	22/40	15
Betaine	118.1	58.0/74.0	90	10	40/29	15
Acetylcholine	146.1	87.0/60.0	100	10	21/15	15
N‐methyl‐d_3_‐acetylcholine (IS)	207.1	85.0/60.0	60	10	28/20	15

Abbreviations: CE, collision energy; CXP, collision‐cell exit potential; DP, declustering potential; EP, entrance potential; eV, electron volts; V, volts.

^a^ The black text corresponds to the mass‐to‐charge (*m/z*) of the quantifying fragment ion, and the red text corresponds to the *m/z* of the qualifying fragment ion.

### 2.13 Targeted LC‐MS/MS method for the tryptophan‐kynurenine pathway

The ion pairing‐based reverse‐phase separation for the measurement of tryptophan‐kynurenine pathway metabolites was performed using a Luna C18(2) and Security Guard C18 analytical and guard column combination described above, and using a MPA solution consisting of water:acetonitrile:formic acid:heptafluorobutanoic acid (99.3:0.5:0.1:0.1, *v:v:v:v*), a MPB solvent consisting of pure acetonitrile, and a NW consisting of acetonitrile:water (1:1, *v:v*). The mobile phase flow rate was 0.100 mL/min, the autosampler trays were chilled to 10°C, the column oven was heated at 50°C, and the gradient elution program used was 0–0.20 min, 5% MPB; 0.2–9.0 min, 5%–80% MPB; 9.0–9.1 min, 80%–5% MPB; 9.1–12.0 min, 5% MPB, with a gradient cycle time of 12.4 min per sample. The retention time for each metabolite was: 3‐hydroxykynurenine, 4.7 min; kynurenic acid, 5.2 min; phenylalanine, 5.5 mins; kynurenine, 5.5 min; and, indole, 7.8 min. A Valco divert valve program was used to divert the chromatographic void to waste during the void elution and column cleanup and equilibration phases of the gradient, and used the following program: 0–0.7 min, valve position B (to waste); 0.7–9 mins, valve position A (to MS system); and, 9.01–12 mins, valve position B (to waste).

A TurboIonSpray® electrospray ionization (ESI) probe was installed in the ionization source and the QTRAP 6500 MS was operated in positive ionization mode using an MRM scan mode with the following instrumental conditions: IonSpray voltage of +5000 V; Cur: 20 psi; Temp: 325°C; GS1/GS2: 25 psi each; CAD gas: HIGH; and Q1/Q3 resolution: Unit/Unit. Table 5 contains the metabolite specific SRM transition parameters for the Tryptophan‐Kynurenine Pathway metabolites.

### 2.14 Targeted LC‐MS/MS method for the trimethylamine N‐oxide (TMAO) pathway method

The hydrophilic interaction chromatography (HILIC) separation for the measurement of TMAO Pathway metabolites was performed using a SeQuant® ZIC‐pHILIC analytical column described above, and using a MPA solution consisting of acetonitrile: water (95:5, *v:v*) and 10 mM ammonium acetate (pH 5.2), a MPB solvent consisting of acetonitrile: water (1:1, *v:v*) and 10 mM ammonium acetate (pH 5.2), and a NW solution consisting of acetonitrile: water (1:1, *v:v*). The mobile phase flow rate was 0.300 mL/min, the autosampler trays were chilled to 4°C, the column oven was heated at 40°C, and the gradient elution program used was 0–2.5 min, 30% MPB; 2.5–9.5 min, 30%–80% MPB; 9.5–12.0 min, 80% MPB; 12.0–12.1 min, 80%–30% MPB; 12.1–16.0 min, 30% MPB, with a gradient cycle time of 16.4 min per sample. The retention time for each metabolite was: TMAO, 10.4 min; free carnitine, 8.8 min; acetylcarnitine, 8.1 min; acetylcholine, 4.6 min; choline, 6.0 min; and betaine, 5.5 min. A Valco divert valve program was used to divert the chromatographic void to waste during the void elution and column cleanup and equilibration phases of the gradient, and used the following program: 0–2.5 min, valve position B (to waste); 2.5–12.0 min, valve position A (to MS system); and 12.1–16 min, valve position B (to waste). An ESI probe was installed in the ionization source and the QTRAP 6500 MS was operated in positive ionization mode using an MRM scan mode with the following instrumental conditions: IonSpray voltage of +5000 V; Cur: 20 psi; Temp: 450°C; GS1/GS2: 30 psi each; CAD gas: HIGH; and Q1/Q3 resolution: Unit/Unit. Table 6 contains the metabolite specific SRM transition parameters for the TMAO Pathway metabolites.

### 2.15 Sample preparations for reverse‐phase nontargeted metabolomics

A 10‐μL volume of each maternal breast milk and infant fecal sample extract was diluted in a 90‐μL volume of a solution consisting of acetonitrile: water (5:95, v:v) with 0.1% formic acid directly into an autosampler vial, and the samples were vortex‐mixed briefly and transferred to an autosampler module, and a 5‐μL sample volume was injected onto the high‐resolution LC‐MS/MS system for bioanalysis.

### 2.16 LC‐MS/MS method for reverse‐phase nontargeted metabolomics

The reverse‐phase chromatographic separation for the nontargeted metabolomics measurement was performed using a Luna C18(2) and Security Guard C18 analytical and guard column combination described above, and using a MPA solution consisting of water that contained 0.1% formic acid, a MPB solution consisting of acetonitrile that contained 0.1% formic acid, and a NW solution composed of a mixture of acetonitrile: water (1:1, v:v). The mobile phase flow rate was 0.080 mL/min, the autosampler tray was chilled to 15°C, and the column oven was heated to 40°C. The gradient elution program was specified as follows: 0–2 min, 5% MPB; 2–25 min, 5%–90% MPB; 25–30 min, 90% MPB; 30–31 min, 90%–5% MPB; and 31–40 min, 5% MPB; and at 40.01 min a Stop command is specified, and there is a 40.4 min duty cycle for each injection.

Nontargeted metabolomics experiments were performed using both positive mode and negative mode acquisitions for the maternal breast milk and infant fecal sample extracts. The heated electrospray (H‐ESI) ionization source parameters for the Orbitrap Fusion MS were specified in the Xcalibur‐based acquisition method as follows: application mode: small molecule; method duration: 39.5 min; ionization mode polarity, positive mode and negative mode; probe spray voltage, static at +3500 V (positive mode) or −2,500 V (negative mode); gas mode, static; sheath gas (arbitrary units), 25; auxiliary gas (arbitrary units), 5; sweep gas (arbitrary units), 0; ion transfer tube temperature, 275°C; and, vaporizer temperature, 75°C. Global MS acquisition parameters were specified as follows: infusion mode, liquid chromatography; expected LC peak width (s), 30; advanced peak determination, false; mild trapping, false; default charge state, 1; enable Xcalibur AcquireX method modification, false; and, internal mass calibration, Easy‐IC™.

The acquisition parameters for the HRMS/HRMS acquisition method used for the reverse‐phase chromatographic method, for both positive and negative mode ionization methods, were specified as follows: detector type, Orbitrap; Orbitrap resolution, 120,000 (at *m/z* 200); mass range, normal; use quadrupole isolation, true; scan range (m/z), 100–650; RF lens (%), 60; AGC target, standard; maximum injection time mode, custom; maximum injection time (ms), 50; Microscans, 1; data type, profile; polarity, positive and negative for each method; source fragmentation, disabled; and, use Easy‐IC™, true. Dynamic exclusion filtering was enabled with the following parameters specified: exclude after *n* times, 1; exclusion duration (s), 6; mass tolerance, ppm; low, 10; high, 10; exclude isotopes, true; perform dependent scan on single charge state per precursor only, false; and, exclude within cycle, true. Apex detection filtering was enabled with the following parameters specified: expected peak width (FWHM, s), 10; desired apex window (%), 30. Intensity threshold filtering was enabled with the following parameters specified: filter type, intensity threshold; intensity threshold, 5.0e4. The ddMS2 OT HCD based MS2 scan function was enabled with the following parameters specified: isolation mode, quadrupole; isolation window (m/z), 2; isolation offset, off; activation type, HCD; collision energy mode, stepped; HCD collision energies (%), 15, 25, and 35; detector type, Orbitrap; Orbitrap resolution, 30,000 (at m/z 200); mass range, normal; scan range mode, auto; AGC target, standard; maximum injection time mode, custom; maximum injection time, 54 ms; Microscans, 1; data type, profile; and, Use Easy‐IC™, true.

### 2.17 Scaffold elements operational parameters and publicly available databases used

Raw data files were imported into Scaffold Elements for final analysis. The Scaffold Elements parameters used for nontargeted metabolomics data analysis, for both positive and negative mode experiments, were specified with the following search parameters: mass range (m/z), 100–650; retention time range, full range; match type, mass only; parent tolerance, 20.0 ppm; fragment tolerance, 0.1 Da; retention time (RT) tolerance, 0.5 min; treat MS1 peak group as single analyte, FALSE; Perform RT alignment, TRUE; perform feature re‐extraction, TRUE; report unknown analytes, FALSE; all analytes have MS2 data, TRUE. Feature finding parameters were specified as follows: noise threshold, 0.1% of max signal; minimum delta scan time, 0.5 s. Positive mode database searches used the following positively charged precursor ions including [M+H−NH_3_]^+^, [M+H]^+^, and, [M+H−H_2_O]^+^. Negative mode database searches used the following negatively charged precursor ions including [M−H−NH_3_]^−^, [M−H]^−^, and [M−H−H_2_0]^−^.

Publicly available databases searched include the following: hmdb_library_elements.libdb; lipidmaps_library_elements.libdb; IST_hr_msms_v20.libdb; and the METLIN_EXPERIMENTAL_v2017.11.06.libdb. Advanced search settings were specified as follows: ID score retention threshold: 0.7; ISF intensity threshold: 0.1 (10% spectrum max intensity); RT reproducibility threshold: 0.75 (75% reproducibility); RT Inclusion threshold: 1.0 s; RT cross‐charge inclusion threshold: 1.0 s; max aligned RT diff: 1 min; max unaligned RT diff: 5 min; ignore experimental MS2s: FALSE. Metabolite score thresholds include: ID score: 0.7; Log10Intensity: 0; reproducibility: 1. No statistical comparisons were performed using Scaffold Elements. All metabolomics data was manually filtered at a Tier 2 level of scrutiny for inclusion into downstream data analysis (Schymanski et al., 2014).

### 2.18 Tier 2 metabolomics data filtering procedures

All metabolomics data contained in the Excel‐based results table were sorted according to their computed MS2 score (highest to lowest), and all metabolite features lacking an acquired MS2 spectrum were removed from the data set. Metabolite features were then sorted by their analyte names, and any metabolite feature designated as a “cluster of metabolite name” was removed from the data set. Metabolite features were sorted by their retention time (RT), and all “pre‐void” early eluting metabolites with RTs less than the computed void time (1.5 min) of the system were removed from the data set. Further, all metabolite features eluting after the gradient max dwell (>31.5 min) were also removed from the data set. Then, publicly available databases were consulted to determine if each metabolite was to be classified as: isobaric bile acids, or isobaric mono‐, di‐, or tri‐saccharides or hexose‐amine sugars that require specialized chromatography for identification—these metabolite features were removed from the data set. All therapeutic drugs, drug metabolites, or exposome‐based environmental compounds (i.e., artificial sweeteners, flame retardants, or plasticizers) were flagged as such and were interrogated individually for possible inclusion in the data set.

The remaining metabolite features are searched within Scaffold Elements for compliance with the following inclusion criteria: (i) database matched based precursor (MS1) ions; (ii) m/z and ion abundance of fragment ions (*n* ≥ 3) match reference spectra; (iii) consistent RTs observed in extracted‐ion current (XIC) chromatogram overlays for all experimental data; and, (iv) good agreement between empirical and reference isotope ratios (Schymanski et al., 2014). All ions that do not satisfy the above exclusion criteria spelled out above are removed from downstream data processing, statistical testing, and volcano‐plot based analyses. These ions were flagged according to the exclusion criteria used to remove them from the dataset, that is, duplicate metabolite, Poor MS2, in source fragment, poor isotopic distribution matching, or background ion.

### 2.19 Statistics

For targeted LC‐MS/MS measurements, metabolite concentrations falling below the lower limit of detection (LOD) of the calibration curve were recorded as zero. Because these values reflect signal levels outside the quantifiable analytical range, they were not imputed or estimated (e.g., LOD/√2), as such substitution would artificially introduce bias into the dataset. The data was graphed in either R or GraphPad Prism software (version 10) (GraphPad Inc.). Statistical analysis was performed with a two‐way ANOVA with the Tukey's multiple comparisons post hoc test. All analyses were corrected for multiple comparisons by controlling the False Discovery Rate. The data are presented as mean ± standard deviation, with *p* < 0.05 (*) considered statistically significant.

## 3 RESULTS

**FIGURE 1:**
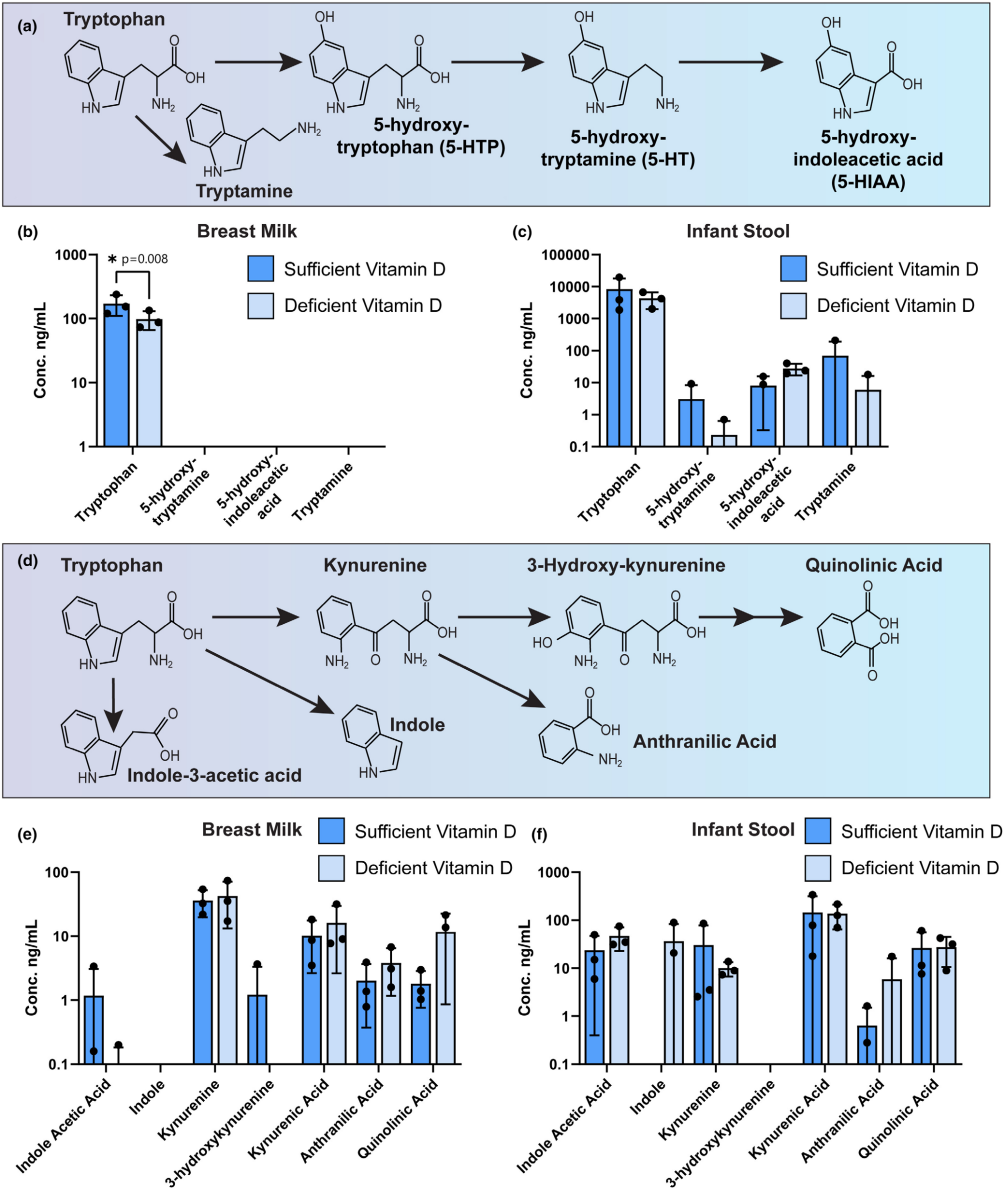
(a) Schematic representation of the chemical pathway involved in the tryptophan to 5‐HIAA pathway. Comparisons of the levels of tryptophan and downstream metabolites between dyads whose breast milk contains sufficient vitamin D (>45 ng/mL) versus deficient vitamin D (<20 ng/mL). Concentrations found in breast milk (b) and infant stool (c) are plotted on a logarithmic scale. (d) Schematic representation of the chemical pathway involved in the tryptophan to quinolinic acid pathway. Concentrations found in breast milk (e) and infant stool (f) are plotted on a logarithmic scale. Each dot represents an individual patient sample (*n* = 3/group). Statistical significance determined by two‐way ANOVA.

Mothers and exclusively breastfeeding infants were enrolled in a randomized 3‐month study designed to determine if maternal vitamin D status was associated with measurable differences in neuroactive compounds in breast milk and infant stool. We first assessed the levels of compounds in the tryptophan pathway by targeted LC‐MS/MS (Figure 1). The amino acid tryptophan is an essential precursor for several neurotransmitters. Tryptophan is converted to 5‐hydroxytryptophan (5‐HTP), which is then converted to the neurotransmitter 5‐hydroxytryptamine (serotonin, 5‐HT), and is eventually metabolized to the waste product 5‐hydroxyindoleacetic acid (5‐HIAA) (Figure 1a). Alternatively, tryptophan can be converted to the neuromodulatory compound tryptamine. We observed tryptophan in all breast milk samples, but serotonin (5‐hydroxy‐tryptamine, 5‐HT), 5‐hydroxyindoleacetic acid (5‐HIAA), and tryptamine were below the detection limit of the measurement (Figure 1b). Breast milk from sufficient vitamin D status mothers contained significantly higher levels of tryptophan (*p* = 0.0082) when compared to milk from mothers with deficient vitamin D status. All four tryptophan‐related compounds were detected in infant stool, but there were no differences in the levels of these compounds based on vitamin D status (Figure 1c).

Tryptophan can also enter the kynurenine pathway to produce neuroactive and immunoregulatory metabolites such as kynurenine, 3‐hydroxykynurenine, quinolinic acid, and anthranilic acid (Figure 1d). Compounds from this pathway were measured in both breast milk and infant stool; however, there were no significant differences based on vitamin D status (Figure 1e,f). Indole and indole‐3‐acetic acid are two additional bioactive downstream products of tryptophan metabolism. Similar to kynurenine pathway metabolites, there were no significant differences in the levels of indole or indole‐3‐acetic acid in the breast milk or infant stool samples based on vitamin D status (Figure 1e,f).

**FIGURE 2:**
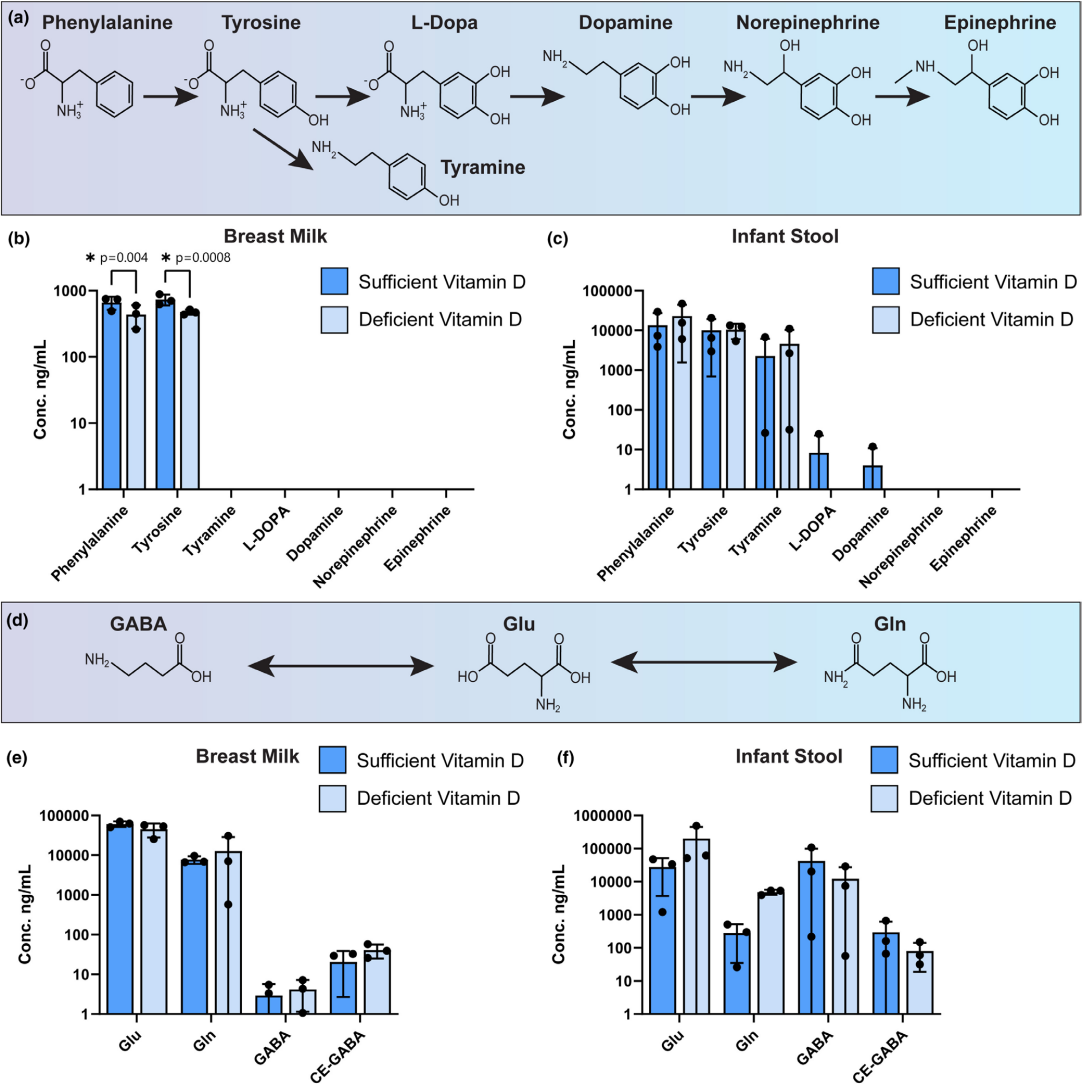
(a) Schematic representation of the chemical pathways involved in tyrosine to epinephrine conversion. Comparisons of the levels of tryptophan and downstream metabolites between dyads whose breast milk contains sufficient vitamin D (>45 ng/mL) versus deficient vitamin D (<20 ng/mL). Concentrations found in breast milk (b) and infant stool (c) are plotted on a logarithmic scale. (d) Schematic representation of the chemical pathway involved in the GABA/Glu/Gln cycles. Concentrations found in breast milk (e) and infant stool (f) are plotted on a logarithmic scale. Each dot represents an individual patient sample (*n* = 3/group). Statistical significance determined by two‐way ANOVA.

Phenylalanine is another essential amino acid in neurotransmitter production. After its conversion to the amino acid tyrosine, it can undergo a series of enzymatic reactions to form levodopa (L‐dopa) and subsequently the neurotransmitters dopamine, norepinephrine, and epinephrine (Figure 2a). Additionally, tyrosine can be metabolized to the sympathomimetic compound tyramine. Interestingly, only phenylalanine and tyrosine were measured in the breast milk samples (Figure 2b). We identified significantly higher levels of phenylalanine (*p* = 0.0037) and tyrosine (*p* = 0.0008) in sufficient vitamin D milk than in deficient vitamin D milk (Figure 2b). In infant stool samples, we measured phenylalanine, tyrosine, tyramine, L‐dopa, and dopamine, but there were no significant differences in the levels of these compounds between the deficient and sufficient vitamin D status groups (Figure 2c).

Another important amino acid is glutamate (Glu), which itself is an excitatory neurotransmitter. Glutamate can also be converted to the inhibitory neurotransmitter gamma‐aminobutyric acid (GABA). GABA can be further modified by the addition of a carboxyethyl‐group, generating gamma‐aminobutyric acid (CE‐GABA). The amino acid glutamine (Gln) additionally serves as a precursor to both Glu and GABA (Figure 2d). Although Glu, Gln, GABA, and CE‐GABA were all detected in breast milk and infant stool samples, there were no significant differences in their levels between deficient and sufficient vitamin D status groups (Figure 2e,f).

**FIGURE 3:**
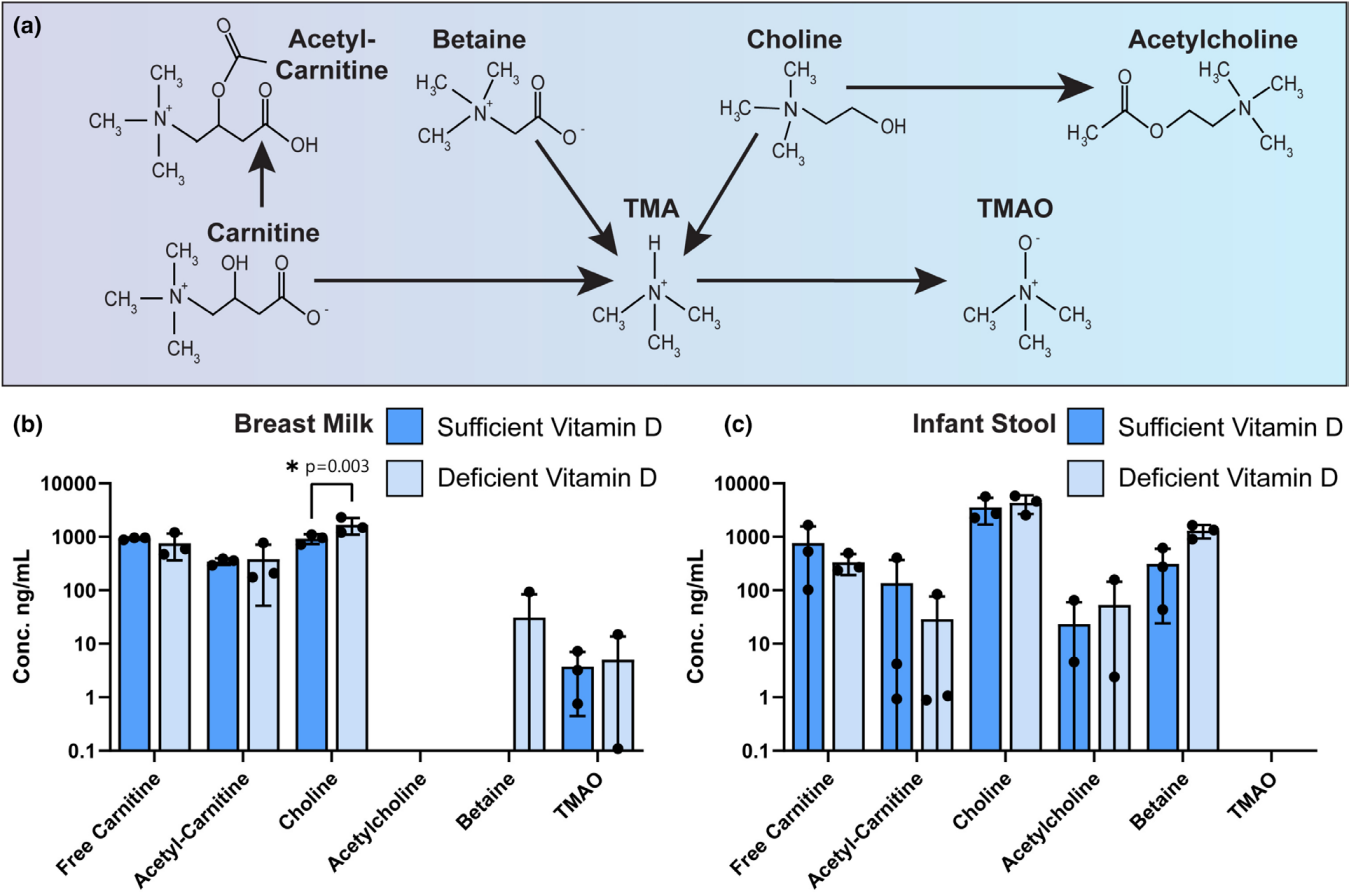
(a) Schematic representation of the chemical pathways involved in trimethylamine‐N‐oxide (TMAO) generation. Comparisons of the levels of tryptophan and downstream metabolites between dyads whose breast milk contains sufficient vitamin D (>45 ng/mL) versus deficient vitamin D (<20 ng/mL). Concentrations found in breast milk (b) and infant stool (c) are plotted on a logarithmic scale. Each dot represents an individual patient sample (*n* = 3/group). Statistical significance determined by two‐way ANOVA.

In addition to neurotransmitters and their related metabolites, we also examined compounds known to affect neuronal processes. We examined choline, L‐carnitine, and betaine (Figure 3a), which have been shown to have neuroprotective roles in the brain (Bhatt et al., 2023; Ferreira & McKenna, 2017; Latham et al., 2021). These three compounds can be converted to the intermediate trimethylamine (TMA) and subsequently to the neuroactive compound trimethylamine‐N‐oxide (TMAO). In a separate series of reactions, carnitine can be conjugated with acetic acid to form acetyl‐carnitine, and choline can be converted into the neurotransmitter acetylcholine. In breast milk we identified carnitine, acetyl‐carnitine, choline, betaine, and TMAO (Figure 3b). Of note, we found that breast milk samples from mothers with deficient vitamin D status contained higher levels of choline (*p* = 0.0034). In the infant stool samples, we detected carnitine, acetyl‐carnitine, choline, acetylcholine, and betaine, but we observed no differences between the vitamin D status groups (Figure 3c).

**FIGURE 4:**
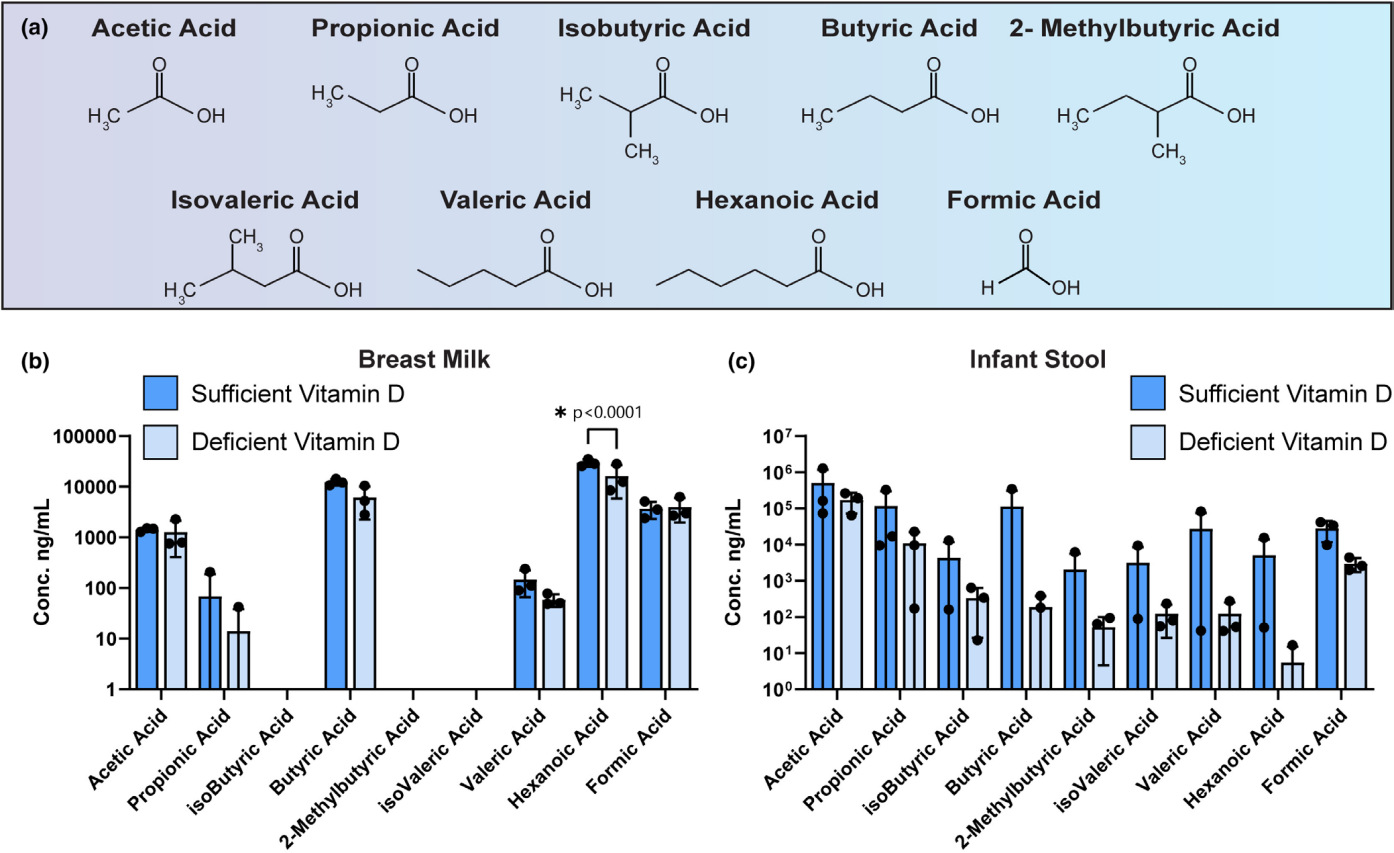
(a) Schematic representation of the chemical structure of short‐chain fatty acids (SCFAs). Comparisons of the levels of SCFAs between dyads whose breast milk contains sufficient vitamin D (>45 ng/mL) versus deficient vitamin D (<20 ng/mL). Concentrations found in breast milk (b) and infant stool (c) are plotted on a logarithmic scale. Each dot represents an individual patient sample (*n* = 3/group). Statistical significance determined by two‐way ANOVA.

Short‐chain fatty acids (SCFAs) are microbially generated metabolites that are speculated to play a crucial role in the gut‐brain axis (Silva et al., 2020). Of the SCFAs, acetic acid (acetate), propionic acid (propionate), and butyric acid (butyrate) are the most well‐studied. However, there is a large variety of SCFAs, including isobutyric acid, modified 2‐methyl‐butyric acid, isovaleric acid, as well as other acids like hexanoic acid, and formic acid (Figure 4a). Reflecting the presence of microbiota in breast milk, we observed acetic acid, propionic acid, butyric acid, valeric acid, hexanoic acid, and formic acid in the milk samples (Figure 4b). We further found that breast milk from sufficient vitamin D status mothers contained higher levels of hexanoic acid (*p* = 0.0001) compared to milk from the deficient vitamin D status group (Figure 4b). We detected all the measured SCFAs in the infant stool samples but identified no differences in SCFA levels between groups (Figure 4c).

**FIGURE 5:**
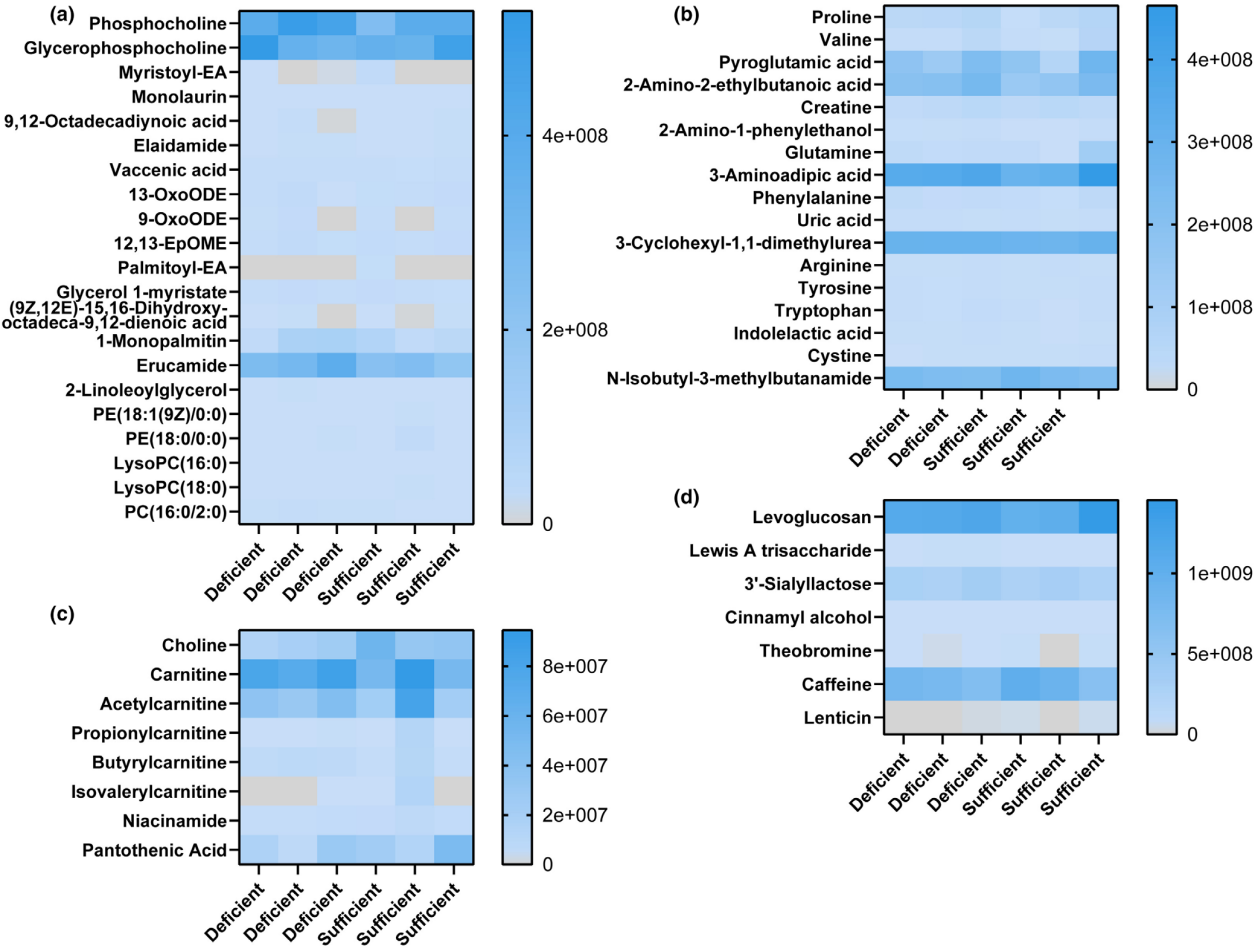
Heat maps of the relative abundance of untargeted metabolomics data from breast milk samples. Metabolites were grouped into the following groups: (a) lipids, (b) amino acids and their derivatives, (c) carnitines and vitamins, and (d) sugars and general metabolites. The higher concentrations are depicted in the dark blue color and values at or close to 0 are depicted in the gray color.

**FIGURE 6:**
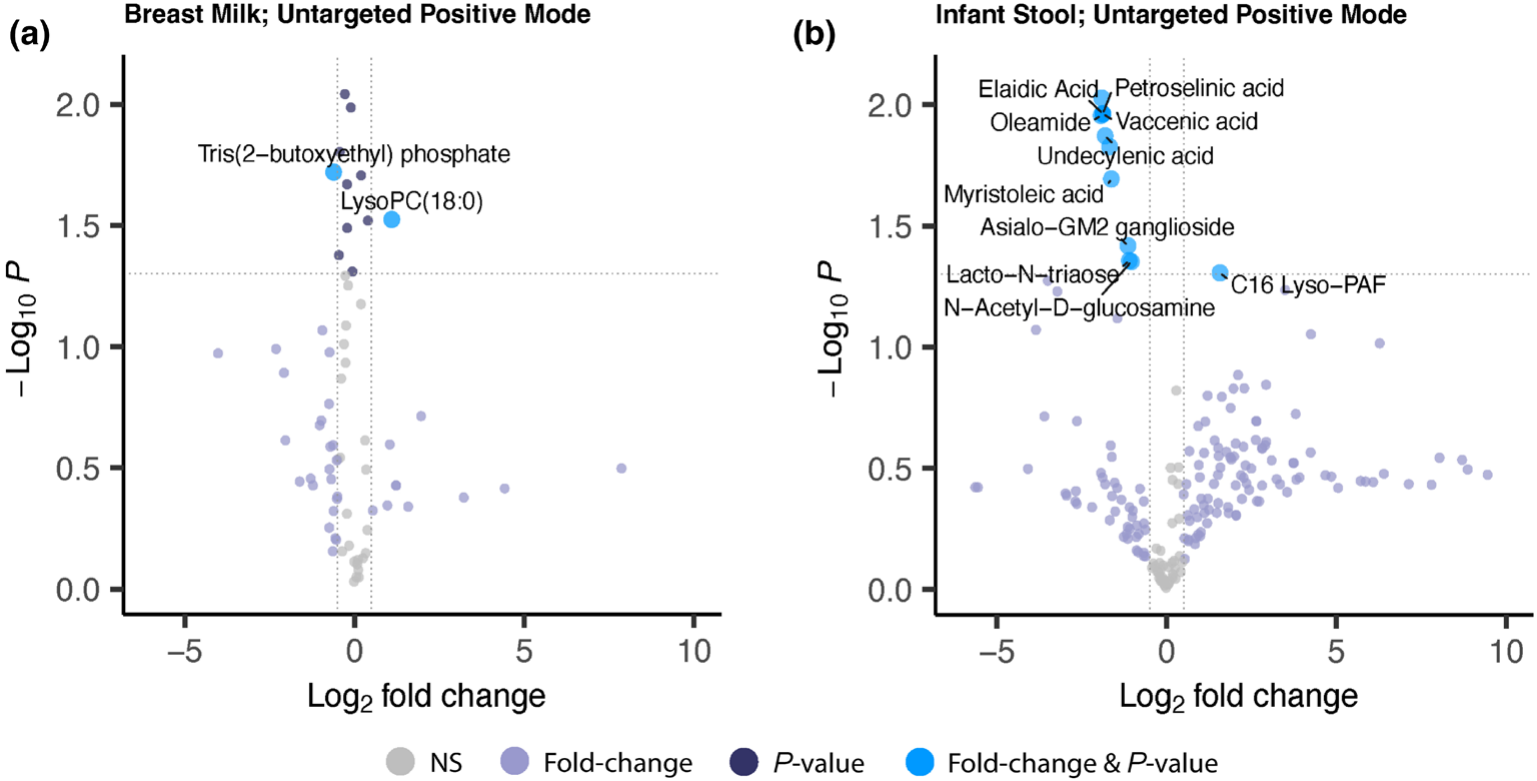
Volcano plot of untargeted metabolomics data illustrating Log_2_ fold‐change differences in (a) breast milk and (b) infant stool metabolite levels in the direction of high to low; that is, a positive fold‐change value indicates that the metabolite levels are higher in the sufficient vitamin D group (>45 ng/mL) compared to the deficient vitamin D group (<20 ng/mL). Significance was determined using dual‐function fold change analyses with t‐tests with MetaboAnalyst, and data were plotted using EnhancedVolcano package in R. Threshold for significance was set to *p* < 0.05, and threshold for Log_2_ fold‐change was set to 0.5.

**FIGURE 7:**
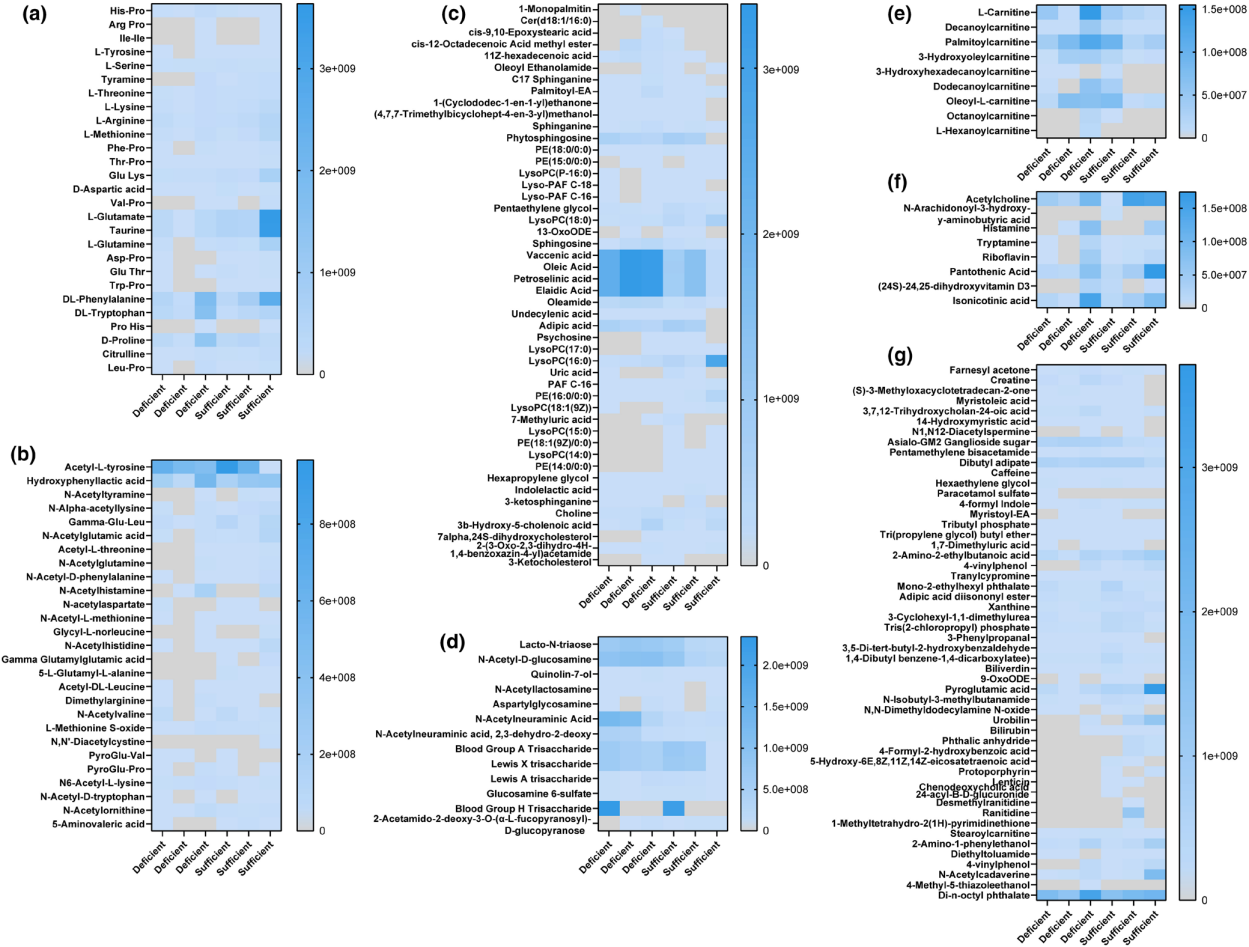
Heat maps of the relative abundance of untargeted metabolomics data from infant stool samples. Metabolites were grouped into the following groups: (a) amino acids, (b) amino acid derivatives, (c) lipids and fatty acids, (d) sugars and quinolones, (e) carnitines, (f) neurotransmitters and vitamins, and (g) general metabolites. The higher concentrations are depicted in the dark blue color and values at or close to 0 are depicted in the gray color.

We identified 69 validated metabolites in the breast milk samples by nontargeted LC‐MS/MS. We observed a variety of lipids (Figure 5a), amino acids and their derivatives (Figure 5b), carnitines and vitamins (Figure 5c), and sugar and general metabolites (Figure 5d) in the breast milk. Among these compounds, we identified 11 different compounds that were elevated in mothers with sufficient vitamin D status (Figure 6). Of interest, we found elevated levels of lysophospholipid LysoPC (18:0) in the milk of mothers with sufficient vitamin D status compared to milk from mothers with deficient vitamin D status (Figure 6a). We also identified 210 validated metabolites in the stool samples. These metabolites included amino acids and their derivatives (Figure 7a,b), lipids and fatty acids (Figure 7c), sugars and quinolones (Figure 7d), modified carnitines (Figure 7e), other neurotransmitters like histamine and acetylcholine, vitamins (Figure 7f), and other metabolites (Figure 7g). In the infant stool, we observed 13 compounds that were differentially regulated based on vitamin D status. Almost all were classified as fatty acids, phospholipids, and sugar derivatives. We observed lower abundance of oleamide, vaccenic acid, lacto‐N‐triaose, and N‐acetyl‐D‐glucosamine in the stool of infants fed milk of mothers with sufficient vitamin D status (Figure 6b). Collectively, all of the data presented indicate that vitamin D status is associated with the levels of select neuroactive compounds, lipids, and sugars in mothers' milk and their infants' stool.

## 4 DISCUSSION

In this study, we assessed neuroactive metabolites in breast milk and infant stool from mothers and exclusively breastfeeding infants, stratified by maternal serum vitamin D status. Targeted LC‐MS/MS analysis revealed that sufficient serum vitamin D status was associated with significantly higher levels of tryptophan, phenylalanine, tyrosine, and hexanoic acid in breast milk. Interestingly, a significantly higher level of choline was measured in breast milk collected from deficient vitamin D status individuals. No significant differences in the kynurenine pathway metabolites, indole derivatives, or glutamate‐GABA pathway compounds were observed between groups. Untargeted metabolomics identified 11 differentially abundant metabolites in sufficient vitamin D milk, including lysophospholipid LysoPC (18:0). In infant stool, neuroactive compounds, including phenylalanine, dopamine, GABA, and short‐chain fatty acids (SCFAs), were detected, but no differences were observed based on maternal vitamin D status. However, untargeted metabolomics identified 13 differentially abundant compounds in infant stool, including oleamide and vaccenic acid. These findings suggest that maternal vitamin D status may be associated with neuroactive compound levels in breast milk and infant stool, which may have implications for infant neurodevelopment.

To our knowledge, we are the first to document the association between maternal vitamin D status with neurotransmitters and their precursors in matched breast milk and infant stool samples. While we saw no direct changes to neurotransmitter levels, we observed higher levels of the amino acid precursors tryptophan, phenylalanine, and tyrosine in the breast milk of mothers with high vitamin D status compared to deficient vitamin D status. Previous studies by Dowlati et al. have also shown that maternal milk levels of the amino acids tryptophan and tyrosine are impacted by dietary supplements (Dowlati et al., 2014, 2015). Their studies, however, were on the short‐term scale (single bolus) and directly supplemented the amino acid, that is, tryptophan and tyrosine. Interestingly, a bolus of the protein alpha‐lactalbumin did not alter levels of the free amino acids. This suggests that amino acid profiles may not be broadly affected by overall dietary protein intake, but other specific dietary components, such as vitamin D, can modulate the milk peptidome. Despite the absence of phenylalanine‐ and tyrosine‐derived neurotransmitters in breast milk and infant stool, it is possible that the higher levels of these amino acids in milk from sufficient vitamin D mothers influences levels of the neurotransmitters in the infant brain. Indeed, Murray et al. showed through radiolabeling that dietary tyrosine not absorbed in the upper digestive tract can indeed reach the gut microbiota and then travel to the brain (Murray et al., 2023). Compared to normal birthweight term age infants, low birthweight infants were found to have lower blood tyrosine and phenylalanine levels (Manta‐Vogli et al., 2020), and it was further reported that lower milk tyrosine levels were associated with slower rates of weight gain in preterm infants (Alexandre‐Gouabau et al., 2019). Infant birthweight and growth are strong predictors of neurodevelopmental outcomes (Pascal et al., 2018; Vohr et al., 2000), and our findings indicate that maternal vitamin D supplementation might indirectly support these factors of the infant condition.

Although we observed several changes in the milk's metabolomic profile, we did not detect corresponding differences in related metabolites in the matched infant stool samples. One possibility is that these amino acids are efficiently absorbed in the infant small intestine, preventing appreciable amounts from reaching the microbiota in the large intestine. Supporting this idea, a study by Schaart et al. (2007) reported that 70%–90% of total protein is absorbed in the small intestine of human neonates, highlighting the infant gut's remarkable capacity for nutrient uptake. The absence of detectable neurotransmitters in the stool could also be due to rapid receptor activation and/or absorption, resulting in levels below the threshold of detection. Alternatively, these precursors may have been utilized by the gut microbiota without being converted into neurotransmitters. For example, *Bifidobacterium* species, which are abundant in breastfed infants, are well versed at using amino acids to support their growth and converting these compounds to aromatic lactic acids (Cui et al., 2022; Engevik et al., 2022; Engevik, Danhof, et al., 2021; Laursen et al., 2021; Waller et al., 2024). Further studies are needed to elucidate the mechanisms underlying this interesting observation.

For targeted analyses of tryptophan metabolism, we assessed levels of select compounds in the kynurenine pathway, which primarily occurs in the liver and is responsible for up to 95% of dietary tryptophan degradation (Badawy, 2017). We also performed targeted analysis of compounds from the indole pathway, which accounts for approximately 5% of dietary tryptophan metabolism and is primarily catalyzed by the gut microbiota (Hou et al., 2023). We observed no differences in these select compounds between the vitamin D status groups. The indole pathway, however, includes many other bioactive derivatives involved in maintaining gut homeostasis such as indole‐3‐lactic acid, indole‐3‐propionic acid, indoleacrylic acid, indole‐3‐aldehyde, and indole‐3‐acetyladehyde (Su et al., 2022). It is possible that levels of these metabolites in milk or infant stool are associated with maternal vitamin D status, but they were not included in our analyses. Similarly, we saw no differences in targeted metabolites of phenylalanine; however, we did not assess levels of other bioactive metabolites involved in neuromuscular and intestinal function such as phenyllactic acid (Oezguen et al., 2022) and phenylpropionic acid (Hu et al., 2023), which were previously found to be elevated in fecal samples from breastfed versus weaned infants (Laursen et al., 2021). Future studies examining these components would be valuable.

Choline, an essential nutrient for infants, was unexpectedly lower in breast milk from mothers of sufficient vitamin D status compared to deficient vitamin D status. Similar to the discussed amino acids, choline serves as a precursor for a neurotransmitter and other bioactive compounds, but we observed no differences in our targeted choline‐derived metabolites. It is known that maternal dietary intake of choline is positively correlated to breast milk choline content (Davenport et al., 2015; Fischer et al., 2010), but it is unclear whether maternal vitamin D intake can directly influence milk choline content or whether the difference in choline levels has any meaningful implications in infant outcomes.

In this study, we identified many SCFAs in our samples. SCFAs are metabolites produced through microbial fermentation and have been implicated in gut–brain communication (Dalile et al., 2019; O'Riordan et al., 2022; Silva et al., 2020). While SCFA production is well‐documented in the large intestine, and one potential source of breast milk SCFAs is absorption from the maternal gut into systemic circulation and subsequent transfer into milk; although this is not the only possible route. Multiple studies have demonstrated that breast milk harbors a distinct resident microbiota, most commonly including *Streptococcus*, *Staphylococcus*, and *Escherichia* species (Kordy et al., 2020; Moossavi et al., 2019; Seferovic et al., 2020; Singh et al., 2023; Xi et al., 2024; Xu et al., 2025). Moreover, the abundance of certain SCFAs, particularly butyrate, has been correlated with specific microbial taxa within breast milk (Xi et al., 2024). We detected higher levels of hexanoic acid in milk from the sufficient vitamin D group compared to the deficient vitamin D group. Known precursors for hexanoic acid production are acetate, lactate, butyrate, and ethanol (Dong et al., 2023). Interestingly, we found that butyrate was trending higher, although not statistically significant (*p* = 0.1341) in milk from sufficient vitamin D mothers when compared to vitamin D deficient mothers. We observed no trends in milk acetate levels, and lactate was not included in our targeted analyses. Vitamin D deficiency is associated with depletion of certain butyrate‐producing bacteria in stool (Robles‐Vera et al., 2019; Velizarova et al., 2023), but it is unknown whether levels of SCFAs or SCFA‐producing microbiota in breast milk are altered according to vitamin status (Zaidi et al., 2021). While we detected no differences in targeted stool SCFAs, our untargeted analyses demonstrated that several medium‐ and long‐chain fatty acids (MCFAs and LCFAs) were elevated in infant stool samples from the deficient vitamin D status group. We were additionally surprised that the human milk oligosaccharide (HMO) lacto‐N‐triaose was elevated in stool from the deficient vitamin D status group. To our knowledge, the influence of maternal nutrient status on MCFA, SCFA, or HMO levels in milk or infant stool has not yet been documented, and more studies are required to address potential implications of these profiles on the infant condition.

Our observations of essentially undetectable levels of dopamine, norepinephrine, and epinephrine are consistent with their extremely short plasma half‐lives of about 2–3 min (PubChem, n.d.; Dalal & Grujic, 2023; Smith & Maani, 2023). The near absence of dopamine and norepinephrine in milk was also confirmed in other studies (Chiba et al., 2019; Ertl et al., 1992). Meanwhile, the neurotransmitters glutamate and GABA, whose half‐lives are on the scale of hours, were detected in almost all milk and stool samples. Relatively high concentrations of GABA in stool as compared to milk are consistent with recent studies suggesting that certain members of the gut microbiota are responsible for generating the majority of circulating GABA levels (Braga et al., 2024). Our group previously found that the microbe *Bifidobacterium dentium* was able to efficiently generate glutamate and GABA, whereas it lacked the cellular machinery to produce dopamine, norepinephrine, or epinephrine (Luck et al., 2021). If these findings in *B. dentium* are applicable to other GABA‐generating microbes, this could further explain the near absence of the other neurotransmitters in our stool samples.

We appreciate that a major limitation is our small sample size (*n* = 3 per group) and that a greater number of samples would enhance the validity of our findings. Furthermore, because investigators remain blinded to treatment assignment in the parent clinical trial of maternal vitamin D supplementation, our classification of infant samples as vitamin D sufficient or deficient does not necessarily reflect the specific supplementation regimen. This limitation is particularly relevant to the analysis of infant stool metabolites, as observed differences could arise from direct vitamin D supplementation to the infant rather than from maternal vitamin D status alone. Nevertheless, these results provide a foundational framework for future studies examining the influence of maternal and infant vitamin D status on infant metabolomic profiles. It would be interesting to perform analyses according to the treatment groups to assess whether the type of supplementation influences outcomes. Additionally, in the future it would be interesting to examine if direct vitamin D supplementation to infants could affect the infant's stool microbiota and metabolites. Given that we only investigated metabolite levels, but not microbial profiles, another potentially insightful avenue is to examine whether the microbiota in breast milk and infant stool are associated with vitamin D status or metabolite levels. Furthermore, significant findings in infant stool were drawn exclusively from our nontargeted analyses; however, to further validate these findings, reassessment of these metabolites through targeted metabolomics with internal standards is warranted. Finally, the most meaningful connection to be studied, through retrospective analyses as well as experimental models, is whether our identified metabolite differences could translate to actual effects on infant development.

In conclusion, we found that maternal vitamin D sufficiency is associated with increases in breast milk levels of the amino acid precursors tryptophan, phenylalanine, and tyrosine. Levels of pathway metabolites or neurotransmitters derived from these amino acids, however, were not significantly different between the vitamin D groups in either milk or infant stool. Other differentially affected metabolites in stool and milk included choline and fatty acids of varying chain lengths, but reasons and implications for these differences remain unclear. Collectively this work provides a more expansive characterization of the impact of maternal vitamin D status on the maternal milk and infant stool metabolome, and we anticipate future studies will uncover meaningful connections between our findings and infant health outcomes.

## AUTHOR CONTRIBUTIONS

Concept and design (ASG, KEC, CLW, and MAE); sample collection and processing (ASG, KEC, TDH, CLW, and MAE); data acquisition (ASG, SJH, KMH, AMH, NO, and TDH); data analysis, statistical analysis, and interpretation (ASG, KEC, ST, JEB, AMH, NO, TDH, CLW, and MAE); drafting the manuscript (ASG); editing manuscript (ASG, KEC, ST, SJH, KMH, JEB, AMH, NO, TDH, CLW, and MAE); obtained funding (MAE).

## FUNDING INFORMATION

This study was supported by a NIH T32DK124191‐01A1 (AG), K01DK123195 (MAE), P30 DK123704 (MAE), P20 GM120457 (MAE), NATS NIH KL2TR001452 (KEC), UL1TR001450 (KEC), S10OD036416 (TDH), P30DK056338 (TDH), and supported by MUSC startup funds to MAE.

## CONFLICT OF INTEREST STATEMENT

TDH is an Editorial Board Member and is contracted as an Associate Academic Editor for Cell Press – STAR Protocols. All other authors have no competing interests to declare.

## Data Availability

The nontargeted metabolomics dataset is publicly available on Metabolomics Workbench under Study ID ST004088 (doi:10.21228/M8TV7M).
